# Editorial: Advanced Strategies for the Plant Protection/Nutrition and Functional Food Production

**DOI:** 10.3389/fnut.2022.955231

**Published:** 2022-06-17

**Authors:** Marko Vinceković, Ivana Pajač Živković, Katarina M. Mikac, Darija Lemić

**Affiliations:** ^1^Department of Chemistry, University of Zagreb Faculty of Agriculture, Zagreb, Croatia; ^2^Department of Agricultural Zoology, University of Zagreb Faculty of Agriculture, Zagreb, Croatia; ^3^Faculty of Science, Medicine and Health, School of Earth, Atmospheric and Life Sciences, University of Wollongong, Wollongong, NSW, Australia

**Keywords:** nutrition, protection, microorganisms, vegetables, biofortification

Excessive use and application of agrochemicals in current agricultural production has had a devastating influence on nature, the environment, and human health, as well as being a source of a variety of pollutants. To limit the use of agrochemicals, it is required to use and apply them in predetermined quantities and with a level of responsibility, while simultaneously beginning to use new approaches in the process of plant nutrition and protection for the development of functional foods. A deeper understanding of the impacts of novel chemicals in plant nutrition and plant protection processes, as well as their market worth, is required to achieve this (1, 2).

One of the most crucial aspects of agriculture and food production is plant protection. With the modernization of agriculture and the introduction of new technologies, old practices of plant protection are no longer sustainable. Plant protection products have therefore contributed to global food security by reducing crop loss, but they have also had a negative influence on the environment and human health (3).

However, certain crop yields would be substantially lowered if plant protection compounds were not available for use in agricultural food production. Darfour and Rosentrater investigated the damage caused by *Sitophilus zeamais* to stored grain in Sub-Saharan Africa, as well as the application of synthetic and botanical pesticides in the Maize protection method. The environmental consequences, ecotoxicity, health impacts, and cost analysis of the active components in Actellic Super (pirimiphos-methyl and permethrin) and NeemAzal (azadirachtin) in the treatment of maize grain were evaluated. Permethrin had the highest ecotoxicity, while pirimiphos-methyl had the lowest. When compared to pirimiphos-methyl, azadirachtin demonstrated no negative effects on human health. When compared to azadirachtin, Actellic Super is 224% less expensive. The costs of both insecticides, however, were determined by the international exchange rate. Regardless of the insecticide employed, the treatment cost increased as grain production increased. Because of the extensive use of insecticides in vegetable production for pest control, some residues remain in vegetables, which can have a negative impact on human health and the occurrence of human diseases (4). The amount of plant protection product used, the time elapsed between the last treatment of the crop and harvest, the number of applications, physicochemical properties, and the crop to which the product is applied all influence the level of these residues (3).

Consequently, it is necessary to develop new compounds and formulations that can be used in the protection of vegetable crops. One of these new insecticides (Indoxacarb) is described in the work of Patra et al. This insecticide is commonly used by vegetable growers in West Bengal, India, to control a wide range of pest insects. The properties of indoxacarb dissipate quickly in the substrates, and the pre-harvest interval was very short. According to their findings, vegetables can be consumed safely a day after the final spraying period. This provides a major promise for future crop protection and time to market including mitigating negative health outcomes for humans and potentially the environment too.

One of the major issues with food on the market is its low nutritional value, which results in a lower intake of minerals and other compounds necessary for normal human biological function. One approach to overcoming this problem was recently addressed by the work of Baldassano et al. who presented the consumption of bio-fortified foods as a way to address the lack of micronutrients. It is estimated that one-third of the world's population lives in areas where iodine is scarce, and a lack of iodine causes a variety of disorders including goiter, reproductive failure, hearing loss, growth impairment, cretinism, and various types of brain injury (1). A cohort of 10 people was supplemented with curly endive leaf biofortified with iodine and 10 subjects with curly endive leaf without biofortification in this short-term double-arm nutritional intervention study. The effects on whole-body homeostasis, specifically iodine, glucose, lipid, hepatic, and iron metabolism, were studied after 12 days. Blood samples were taken at the start and after 12 days of curly endive supplementation, and they were compared to controls. The findings revealed that a short-term iodine curly endive intervention had no effect on whole-body homeostasis in healthy people, but that there was an increase in iodine concentration in urine samples and an increase in vitamin D, calcium, and potassium concentration in blood samples only in the urine samples.

Nutrient element content in food is determined by the amount of nutrients (micro-and macronutrients) that the crop can absorb (5, 6).

Iron is a trace element that plants require in small amounts, but deficiencies can have a significant impact on plant yield and quality. It is a major limiting factor in plant growth. Hence, why it is critical to make iron available and absorbable as easily as possible; and this can be accomplished through the use of microorganisms (4, 7).

Recently, Styczynski et al. investigated Antarctic bacterial strains that are cold activated and act as iron chelators, e.g., *Pseudomonas sp. ANT H12B, Psychrobacter sp. ANT H59*, and *Bacillus* sp. *ANT WA51*. The ability of these strains to promote plant growth was revealed through the production of various biomolecules, such as biosurfactants and siderophores, which increased the availability of nutrients in the environment, neutralized fungal pathogens and their metabolites, and increased the bioavailability of iron in soil by up to 40%.

In conclusion, the findings of the studies discussed above constitute a significant amount of new data and information that might be employed as novel tools and strategies in plant protection/nutrition and functional food production processes. Despite all of the existing literature and evidence related to this extremely important topic, the published papers show that there are still many aspects of plant nutrition/protection and functional food production that need to be investigated and understood through the application of new technologies and strategies that could increase the production and quality of global food resources.

## Author Contributions

All authors wrote sections of the manuscript. All authors listed have made a substantial, direct, and intellectual contribution to the work and approved it for publication.

## Conflict of Interest

The authors declare that the research was conducted in the absence of any commercial or financial relationships that could be construed as a potential conflict of interest.

## Publisher's Note

All claims expressed in this article are solely those of the authors and do not necessarily represent those of their affiliated organizations, or those of the publisher, the editors and the reviewers. Any product that may be evaluated in this article, or claim that may be made by its manufacturer, is not guaranteed or endorsed by the publisher.
